# Developmental Changes in the Magnitude of Representational Momentum Among Nursery School Children: A Longitudinal Study

**DOI:** 10.3389/fpsyg.2022.882913

**Published:** 2022-06-30

**Authors:** Shiro Mori, Hiroki Nakamoto, Nobu Shirai, Kuniyasu Imanaka

**Affiliations:** ^1^Faculty of Physical Education, National Institute of Fitness and Sports in Kanoya, Kanoya, Japan; ^2^Department of Psychology, Faculty of Humanities, Niigata University, Niigata, Japan; ^3^Department of Health Promotion Sciences, Tokyo Metropolitan University, Tokyo, Japan

**Keywords:** representational momentum, developmental changes, nursery school children, longitudinal design, pointing task, judging task

## Abstract

Representational momentum (RM) is a well-known phenomenon that occurs when a moving object vanishes suddenly and the memory of its final or vanishing position is displaced forward in the direction of its motion. Many studies have shown evidence of various perceptual and cognitive characteristics of RM in various daily aspects, sports, development, and aging. Here we examined the longitudinal developmental changes in the displacement magnitudes of RM among younger (5-year-old) and older (6-year-old) nursery school children for pointing and judging tasks. In our experiments, the children were asked to point at by their finger (pointing task) and judge the spatial location (judging task) of the vanishing point of a moving stimulus. Our results showed that the mean magnitudes of RM significantly decreased from 5- to 6-year-old children for the pointing and judging tasks, although the mean magnitude of RM was significantly greater in the 5-year-old children for the pointing task but not for the judging task. We further examined the developmental changes in RM for a wide range of ages based on data from the present study (5-year-old children) and our previous study (7- and 11-year-old children and 22-year-old adults). This *ad hoc* examination showed that the magnitude of RM was significantly greater in 5-year-old children than in adults for the pointing and judging tasks. Our findings suggest that the magnitude of RM was significantly greater in young children than in adults and significantly decreased in young children through adults for the pointing and judging tasks.

## Introduction

The concept of representational momentum (RM) was first reported by Freyd (1983, 1987) and Freyd and Finke (1984). Freyd hypothesized that the mental representation of a moving object in a memory system is generally dynamic rather than static. This was demonstrated in their experiments, which showed a forward displacement of the memory representation of the final position in a series of rectangular pictures presented at an increasing angle of orientation, inducing an apparent rotary motion of the rectangular pictures (Freyd and Finke, 1984). Hubbard and Bharucha (1988) used the linear movement of a dot stimulus to further examine the nature of RM. In their experiments, the moving dot stimulus suddenly vanished, and the participants were asked to spatially judge its vanishing point. The participants tended to judge the vanishing point slightly forward of the dot’s position rather than the actual/physical vanishing point (Hubbard, 2005, 2015, 2017, for review).

Many previous studies have intensively investigated the nature of RM and reported that it is a function of various perceptual and cognitive characteristics such as stimulus velocity (Finke et al., 1986; Getzmann and Lewald, 2009; Feinkohl et al., 2014; Merz et al., 2019), eye movements (Kerzel, 2000, 2006), and attention (Hayes and Freyd, 1995; Kerzel, 2003; Hubbard et al., 2009). Furthermore, RM may develop through relatively long-term learning and experience, resulting in a greater magnitude of RM characterized by sports experts (Gorman et al., 2011, 2012; Nakamoto et al., 2015; Jin et al., 2017; Anderson et al., 2019; Chen et al., 2021) and professionals of other fields, such as aircraft pilots (Blättler et al., 2011) and automobile drivers (Blättler et al., 2010, 2012). Furthermore, the developmental (Futterweit and Beilin, 1994; Hubbard et al., 1999; Perry et al., 2008; Shirai et al., 2018) and aging (Piotrowski and Jakobson, 2011) aspects of RM have also been examined. Previous studies provided robust empirical evidence of the perceptual, cognitive, and behavioral nature of RM.

Regarding the developmental aspects of RM, several studies reported that it develops in the early developmental stages. Perry et al. (2008) reported that toddlers aged 2–3 years showed perceptual judgments indicative of RM and suggested that it may develop in the early growth stage in toddlers. Hubbard et al. (1999) showed that the magnitude of RM measured in a pointing task was greater in school-aged children than in adults. Futterweit and Beilin (1994) examined the magnitude of RM in a judging task and found no significant differences between school-aged children and adults. The findings of Hubbard et al. (1999) and Futterweit and Beilin (1994) on RM in school-aged children and adults are controversial; however, different tasks were used in these studies.

Regarding previous equivocal findings of the developmental aspects of RM in school-aged children and adults, our previous study (Shirai et al., 2018) examined RM among younger and older school-aged children and adults and showed that the effect of age on the magnitude of RM was significant in the pointing task and somewhat modest in the judging task. This supports the findings of the pointing task reported by Hubbard et al. (1999) and the judging task reported by Futterweit and Beilin (1994). Hence, the magnitude of RM may be greater in children than in adults, although this was typically evident in the pointing task and modest in the judging task. If the magnitude of RM is generally greater in children than in adults, it can be assumed that RM develops during early childhood and gradually deteriorates with age. If this assumption is true, the developmental nature of RM might be innate; alternatively, RM develops in the early stages of childhood rather than gradually through adulthood.

To elucidate the developmental aspects of RM in much younger children, we examined the developmental features of nursery school children through experiments using pointing and judging tasks. Previous controversial findings of the magnitudes of RM in school-aged children versus adults might have resulted from large individual differences among children; hence, we evaluated both younger and older children using a longitudinal study design to minimize individual differences. Children who participated in this study were evaluated twice in two successive years in the younger and older nursery school classes. The methods (tasks, procedures, and data analyses) used were similar to those used in the Shirai et al. (2018) study except for the longitudinal study design and participant age. The range of participant age in the present study was 5.4–6.4 years, whereas that in the Shirai et al. (2018) study was 7.4–10.8 years and 22-year-old adults. Furthermore, the procedures of the two studies differed slightly in terms of number of trials and order of the tasks (see the Methods section). First, we investigated longitudinal developmental changes in the magnitude of RM in 5–6-year-old children. Second, we compared the results of the present study with those reported by Shirai et al. (2018) and discussed the overall developmental changes in RM in a wide range of children as well as adults.

## Materials and Methods

### Participants

A total of 31 children^1^ (16 boys, 15 girls) participated in this longitudinal study. All participants were evaluated twice (in March of two successive years) when in the younger and older nursery school classes [mean age, 5.4 (SD, 0.27 years) and mean age, 6.4 years (SD, 0.27 years), respectively]. They were labeled as 5- and 6-year-old children, respectively. Among the 31 children, two boys and three girls did not perform well in the pointing and/or judging task with extremely low/high RM scores (beyond 3 SD from respective group means) or failed to complete the scheduled trial sessions because of boredom or tiredness. These individuals were excluded from the subsequent analyses. Thus, 26 children (14 boys, 12 girls) were included in the analysis. All of the children had normal or corrected-to-normal vision and no reported history of any visual/motor disorders. Written informed consent was obtained from all of the children’s parents. This study was approved by the Ethics Committee of the National Institute of Fitness and Sports in Kanoya, Kanoya, Japan and conducted in accordance with the code of ethics of the Declaration of Helsinki and its future amendments.

### Apparatus

The experiments were conducted in a separate room to isolate the participants from visual and auditory noises other than the experimental stimuli. The participants sat comfortably on a chair and faced a 27-inch touch-sensitive liquid crystal display (LCD) screen (ProLite T2735MSC-B1; 1,920 × 1,080 pixels, 60 Hz, 597.6 × 336.2 mm presentation field; Iiyama, Inc., Japan). The 27-inch touch-sensitive LCD screen was used to present visual stimuli for the pointing and judging tasks and retrieve the participants’ responses (finger pointing on the 27-inch touch-sensitive LCD screen) in the pointing task. In the judging task, participants’ responses (verbal response, “forward” and “backward”) were recorded by the experimenter on a personal computer system (*CF*-AXNEABRFEPTU; Panasonic Inc., Japan). Both processes, presenting stimuli and recording the participants’ responses, were performed using a software program created by author KI with a presentation software package (version 17.1; Neurobehavioral Systems, Inc., USA) on a personal computer system.

### Visual Stimuli

A black-colored bear-like cartoon character (widely known to children and adults in Japan) was used as a visual stimulus; it was exactly the same size in pixels (but not in visual angle)^2^ as that used in our previous study (Shirai et al., 2018). In each trial, to capture participants’ attention with the visual stimulus, a large-sized cartoon character [465 pixels (22.8°) in width, 644 pixels (31.3°) in height] first appeared at the center of the white presentation field of the 27-inch touch-sensitive LCD screen. The cartoon character’s upper limbs moved up and down according to the rhythmic high and low tone sounds emitted twice for 1,900 ms. Thereafter, the size of the cartoon character was rapidly reduced to approximately 84% of its original size [390 pixels (19.1°) in width, 535 pixels (26.2°) in height], and the cartoon character moved toward a position 500 pixels (24.5°) away from the right or left (randomly selected in each trial) edge. After a randomized interval ranging from 500 to 1,000 ms, the size of the cartoon character was reduced to approximately 17% of its original size [78 pixels (3.8°) in width, 105 pixels (5.2°) in height]; and the cartoon character moved toward the other side. The moving speed of the cartoon character was accelerated from 0 to 30 pixels per frame (82.3°/s) in the first six frames (0.1 s) and maintained until the cartoon character reached the end position. The distance between the initial and end positions was altered randomly in the range of 460–920 pixels (22.5°–45.0°). After reaching the end position, the cartoon character either immediately vanished (immediate-vanishing condition) or remained stationary for 500 ms and then vanished (delayed-vanishing condition). Both the immediate- and delayed-vanishing conditions were used to collect displacement data to calculate the magnitude of RM using the subtraction method, the details of which are described in the last part of the “Materials and Methods” section.

### Procedures

The participants sat on a chair in front of a 27-inch touch-sensitive LCD screen with no head or chin rests. The experimenter, a graduate student, was paid to conduct the experiments and sat in front of a personal computer system placed on the right side of the screen. At the beginning of the experimental trial session, the experimenter adjusted the viewing distance between the participant and the 27-inch touch-sensitive LCD screen to the distance where the participant would be able to touch the 27-inch touch-sensitive LCD screen. This resulted in an average of approximately 30–42 cm (at the center and the edge of the screen, respectively). Each participant performed the pointing and judging tasks under immediate- and delayed-vanishing conditions. The pointing task was followed by the judging task, with the delayed-vanishing condition followed by the immediate-vanishing condition. Fixed trial orders (instead of the counterbalanced order) of tasks and vanishing conditions were used because the pointing task and delayed-vanishing condition were much easier to perform than the judging task and immediate-vanishing condition, respectively, particularly for the 5-year-old children. Based on the data of Shirai et al. (2018), the effects of trial orders^3^ on the magnitude of RM were investigated later. No significant effect of trial order on the magnitude of RM was found in the younger and older children. The pointing and judging tasks under the immediate- and delayed-vanishing conditions were completed within 20–30 min, including short rests between experimental sessions.

In the pointing task, the cartoon character moved to the left- or right-end position and vanished immediately (the immediate-vanishing condition) or vanished with a 500-ms delay (the delayed-vanishing condition). The participants were asked to touch the screen with their index finger at the perceived vanishing point. The displacement along the horizontal axis between the touched position and the actual vanishing point was recorded as the pointing error. Each participant performed 20 trials of the pointing task for each vanishing condition (immediate- and delayed-). Hence, 40 trials were performed of the pointing task. The pointing task for each vanishing condition typically lasted for approximately 5–6 min. The mean pointing error was calculated based on 20 pointing errors for the immediate- and delayed-vanishing conditions.

In the judging task, the cartoon character moved to the end position and vanished immediately (the immediate-vanishing condition) or vanished with a 500-ms delay (the delayed-vanishing condition), similar to that in the pointing task. Unlike the pointing task, after a 400-ms interval, the cartoon character again appeared as a probe stimulus at a location either forward (+ shift) or backward (− shift) by a certain distance (step size) from the end or the vanishing point (defined as 0). The participant was asked to report their judgment regarding the probe location relative to the vanishing point either verbally as “forward” or “backward” or by hand or finger in the left or right direction, which corresponded to forward or backward when they had difficulty reporting their judgment verbally. The participant’s report was then recorded by the experimenter on the computer system, and the probe stimulus then disappeared. We used the staircase method to measure the point of subjective equality (PSE) for individual judgments of the end/vanishing point. In each trial, the location of the probe stimulus was further shifted by a step size forward or backward from the end or vanishing point based on the participant’s judgment in the immediately preceding trial. If the participant made a judgment of “backward,” the location of the probe stimulus was shifted by a step size in the *forward* direction in the next trial and vice versa. In the case the participant’s judgment altered from “backward” to “forward” or vice versa, the direction (forward/backward) of the probe stimulus in the next trial was reversed.

At the beginning of the staircase session, the probe stimulus location was set 400 pixels (19.6°) backward from the vanishing position. This was because we assumed that all participants would respond correctly (i.e., report a “backward” judgment) that the location of the probe stimulus was shifted backward by a large (−400 pixels) shift from the vanishing point. The initial step size was set to 200 pixels (9.8°), and the step size was decreased by half at each reverse point until the step size reached 25 pixels (1.3°), which was maintained until the end of the staircase session. Each staircase session continued until the direction in which the probe stimulus was shifted was reversed 12 times, which lasted approximately 5–10 min differing among the participants. Individual PSE was calculated as the mean of the location (relative to the vanishing point in pixels) of the probe stimulus over the last 10 reversal points.

### Subtraction Method Used to Calculate the Magnitude of RM

We used the subtraction method (Shirai et al., 2018) to calculate the individual magnitudes of RM. In this method, the mean displacements (i.e., pointing errors for the pointing task and PSE for the judging task) in the delayed-vanishing condition are subtracted from those in the immediate-vanishing condition. In the immediate-vanishing condition, the moving stimulus suddenly vanished at the endpoint of the stimulus run, whereas in the delayed-vanishing condition, the stimulus remained stationary for 500 ms at the endpoint; thereafter, the stimulus vanished. In the delayed-vanishing condition, no RM should occur because the stimulus was stationary for 500 ms at the endpoint before the stimulus vanished, resulting in a still, rather than moving, stimulus at the last stage. In contrast, in the immediate- and delayed-vanishing conditions, some likely individual biases irrelevant to RM may occur in the pointing and/or judgment responses. Therefore, the resultant displacements in the delayed-vanishing condition can be considered a baseline (because of no RM but individual biases) for either the individual pointing or judgment responses to estimate the true magnitudes of RM without individual biases.

## Results

### Displacements in Pointing and Judging Tasks Under Immediate- and Delayed-Vanishing Conditions

**Figure 1:**
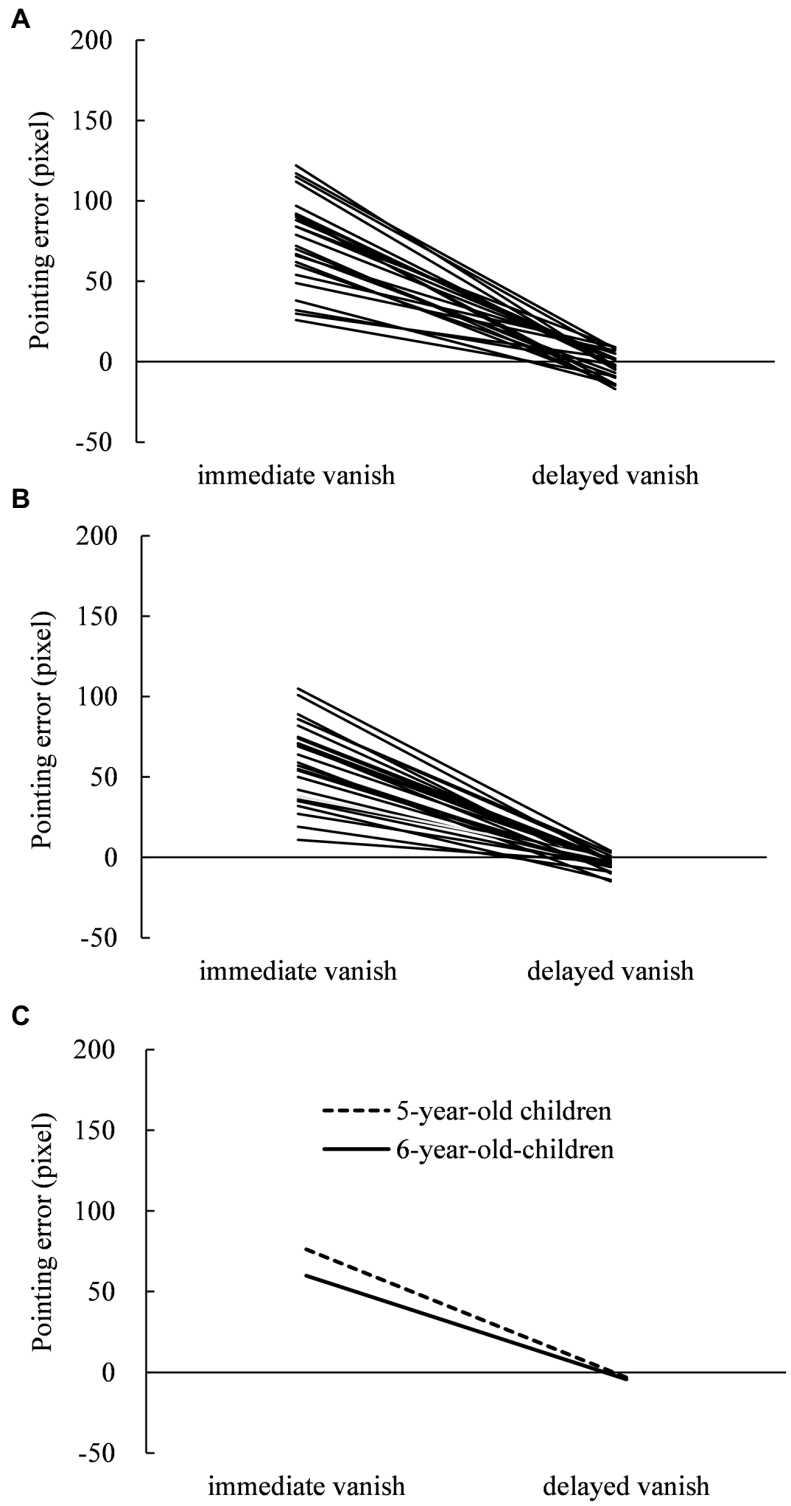
Individual pointing errors (displacement between the pointed position and actual vanishing point) in the pointing task under the immediate- and delayed-vanishing conditions in **(A)** 5-year-old children and **(B)** 6-year-old children with **(C)** mean pointing errors for both.

The displacements were measured with respect to pointing errors in the pointing task and PSE in the judging task under the immediate- and delayed-vanishing conditions. In the pointing task, individual pointing errors in 5- and 6-year-old children are shown in Figures 1A,B, respectively, while their means are shown in Figure 1C. Most 5- and 6-year-old children showed larger displacements in the immediate-vanishing condition than in the delayed-vanishing condition. A two-way repeated measures ANOVA performed on the pointing error data showed a significant main effect of vanishing condition (*F*_1,25_ = 257.064; *p* < 0.001; $\eta_{p}^{2}$ = 0.911) and age (*F*_1,25_ = 11.258; *p* < 0.003; $\eta_{p}^{2}$ = 0.311), and the interaction between the two factors was also significant (*F*_1,25_ = 11.467; *p* = 0.002; $\eta_{p}^{2}$ = 0.314).

Subsequent simple main effects tests showed that the simple main effects of the vanishing condition were significant in both 5-year-old (*F*_1,25_ = 220.571; *p* = 0.0001; $\eta_{p}^{2}$ = 0.898) and 6-year-old (*F*_1,25_ = 192.555; *p* = 0.0001; $\eta_{p}^{2}$ = 0.885) children, whereas the simple main effect of age was significant in the immediate-vanishing condition (*F*_1,25_ = 12.855; *p* = 0.001; $\eta_{p}^{2}$ = 0.340), but not in the delayed-vanishing condition (*F*_1,25_ = 0.499). One-sample t-tests showed that the mean pointing errors in the immediate-vanishing condition were significantly larger than 0 for both 5-year-old (mean, 76.2 pixels; SD = 27.12; *t*_25_ = 14.318; *p* < 0.001; Cohen’s *d* = 2.808) and 6-year-old children (mean, 59.9 pixels; SD = 24.80; *t*_25_ = 12.320; *p* < 0.001; Cohen’s *d* = 2.416). Furthermore, the mean pointing errors in the delayed-vanishing condition showed a nearly significant difference from 0 for 5-year-old children (mean, −3.1 pixels; SD = 7.92; *t*_25_ = −2.007; *p* = 0.056; Cohen’s *d* = −0.394) and a clearly significant difference from 0 for 6-year-old children (mean, −4.3 pixels; SD = 4.54; *t*_25_ = −4.837; *p* < 0.001; Cohen’s *d* = −0.949).

**Figure 2:**
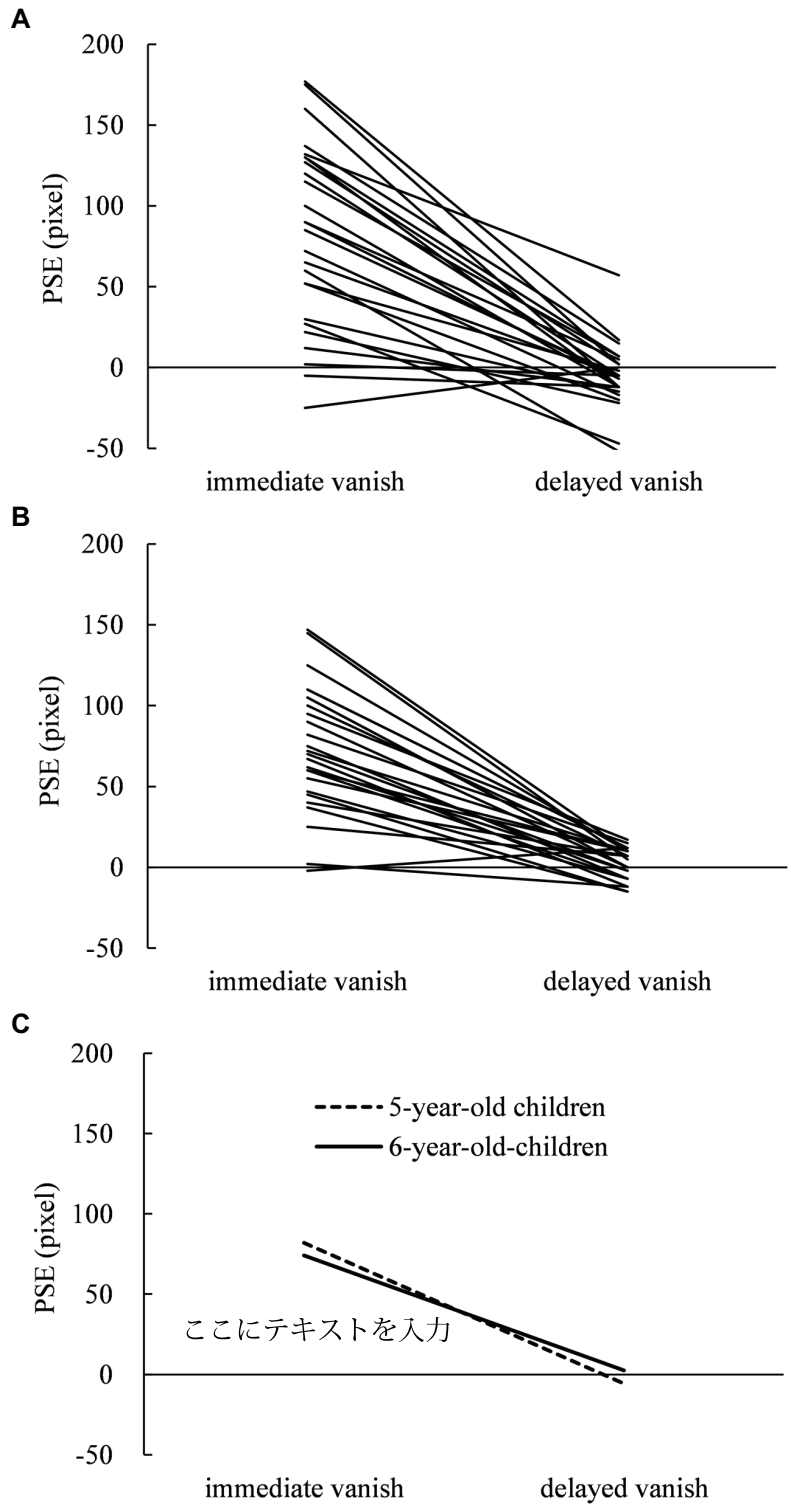
Individual points of subjective equality (PSEs) in the judging task under the immediate- and delayed-vanishing conditions in **(A)** 5-year-old children and **(B)** 6-year-old children with **(C)** mean PSEs for both.

For the judging task, individual PSE in 5- and 6-year-old children are shown in Figures 2A,B, respectively, while their means are shown in Figure 2C. Most 5- and 6-year-old children showed larger displacements in PSE in the immediate-vanishing condition than in the delayed-vanishing condition. A two-way repeated-measures ANOVA performed on the PSE data showed that the main effect of the vanishing condition was significant (*F*_1,25_ = 118.340; *p* < 0.001; $\eta_{p}^{2}$ = 0.826), but that of age was not significant (*F*_1,25_ = 0.002). Furthermore, the interaction between these two factors was not significant (*F*_1,25_ = 3.016; *p* = 0.095; $\eta_{p}^{2}$ = 0.108). Simple main effects of the vanishing condition were significant for 5-year-old (*F*_1,25_ = 77.012; *p* < 0.0001; $\eta_{p}^{2}$ = 0.755) and 6-year-old children (*F*_1,25_ = 102.269; *p* < 0.0001; $\eta_{p}^{2}$ = 0.804). One-sample t-tests showed that the mean PSE in the immediate-vanishing condition was significantly larger than 0 for both 5-year-old (mean, 82.0 pixels; SD = 57.00; *t*_25_ = 7.335; *p* < 0.001; Cohen’s *d* = 1.439) and 6-year-old children (mean, 74.2 pixels; SD = 38.65; *t*_25_ = 9.788; *p* < 0.001; Cohen’s *d* = 1.920), whereas the mean PSE in the delayed-vanishing condition did not show any significant difference from 0 for 5-year-old (mean, −5.8 pixels; SD = 20.21; *t*_25_ = −1.455; *p* = 0.158; Cohen’s *d* = −0.285) or 6-year-old children (mean, 2.5 pixels; SD = 9.69; *t*_25_ = 1.315; *p* = 0.200; Cohen’s *d* = 0.258).

### Magnitudes of RM for the Pointing and Judging Tasks in 5- and 6-Year-Old Children

The magnitudes of the individual RMs were calculated by subtracting the displacements (pointing errors for the pointing task and PSEs for the judging task) in the delayed-vanishing condition from those in the immediate-vanishing condition.

**Figure 3:**
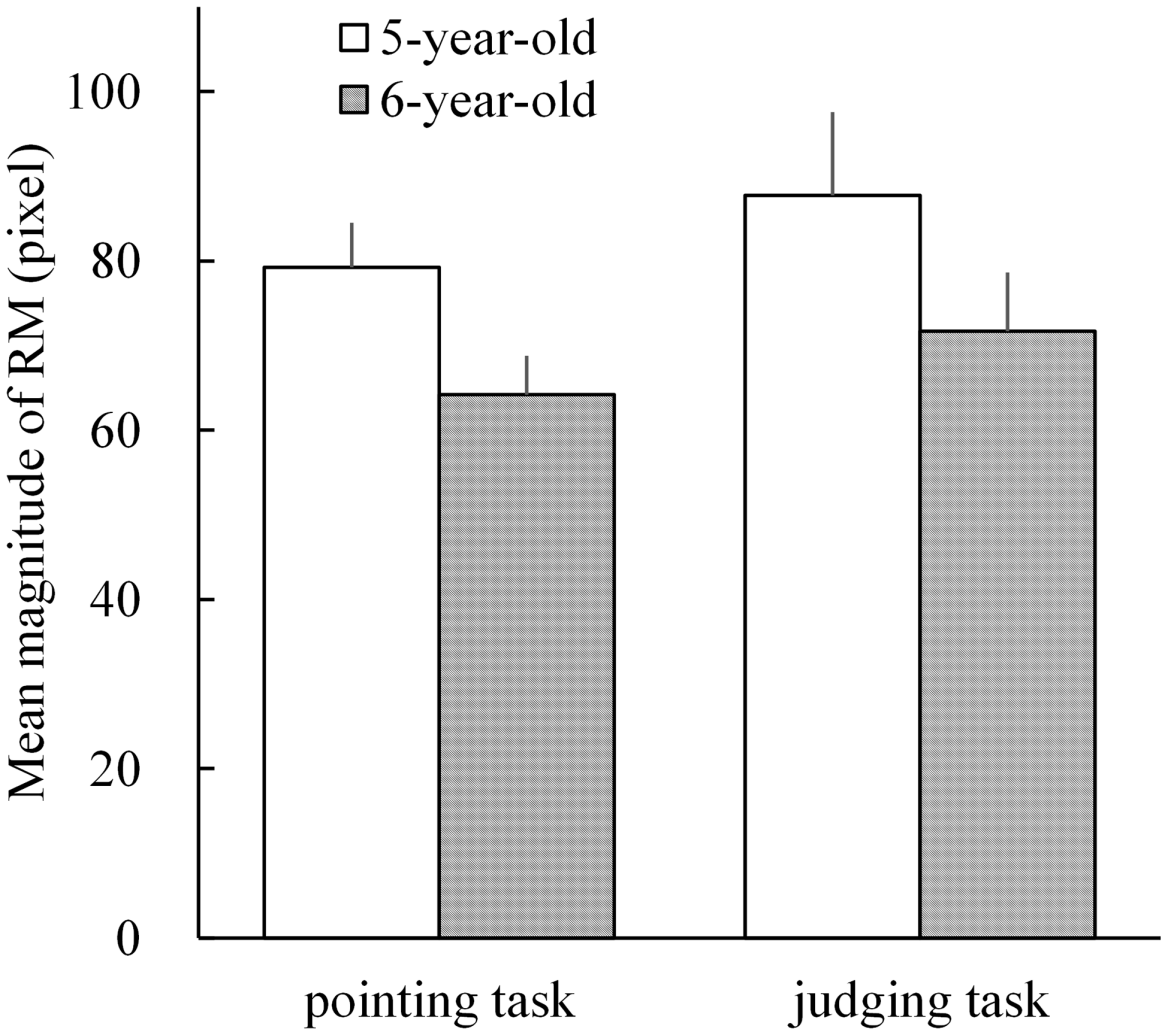
The mean magnitudes of representational momentum (RM) in 5- and 6-year-old children for the pointing and judging tasks. Individual magnitude of RM was calculated by subtracting the individual mean displacements (pointing errors in the pointing task; and points of subjective equality, PSE, in the judging task) under the delayed-vanishing condition from those in the immediate-vanishing condition. The error bars present 1 standard error.

The mean magnitudes of RM in 5- and 6-year-old children for both pointing [mean = 79.3 pixels (SD = 27.2) and mean = 64.2 pixels (SD = 23.6), respectively] and judging tasks [mean = 87.8 pixels (SD = 51.0) and mean = 71.7 pixels (SD = 36.2), respectively] are shown in Figure 3. All mean magnitudes of RM were significantly larger than 0 (*t*_25_ = 8.776 to 14.852; *p* < 0.001 for all; Cohen’s *d* = 1.721 to 2.913). A repeated measures ANOVA on the mean magnitudes of RM was performed with two factors: age (5- and 6-year-old children) and task (pointing and judging tasks). The ANOVA results showed a significant main effect of age (*F*_1,25_ = 10.066; *p* = 0.004; $\eta_{p}^{2}$ = 0.287) but not of task (*F*_1,25_ = 1.209; *p* = 0.282; $\eta_{p}^{2}$ = 0.046) or no interaction between these two factors (*F*_1,25_ = 0.009). Although the interaction was not significant, Shirai et al. (2018) reported that the age effect on the magnitude of RM varied based on pointing and judging tasks. Therefore, an additional simple main effect analysis was conducted. The results showed that the simple main effect of age was significant for the pointing task (*F*_1,25_ = 11.467; *p* = 0.002; $\eta_{p}^{2}$ = 0.314) but not for the judging task (*F*_1,25_ = 3.016; *p* = 0.095; $\eta_{p}^{2}$ = 0.108).

**Figure 4:**
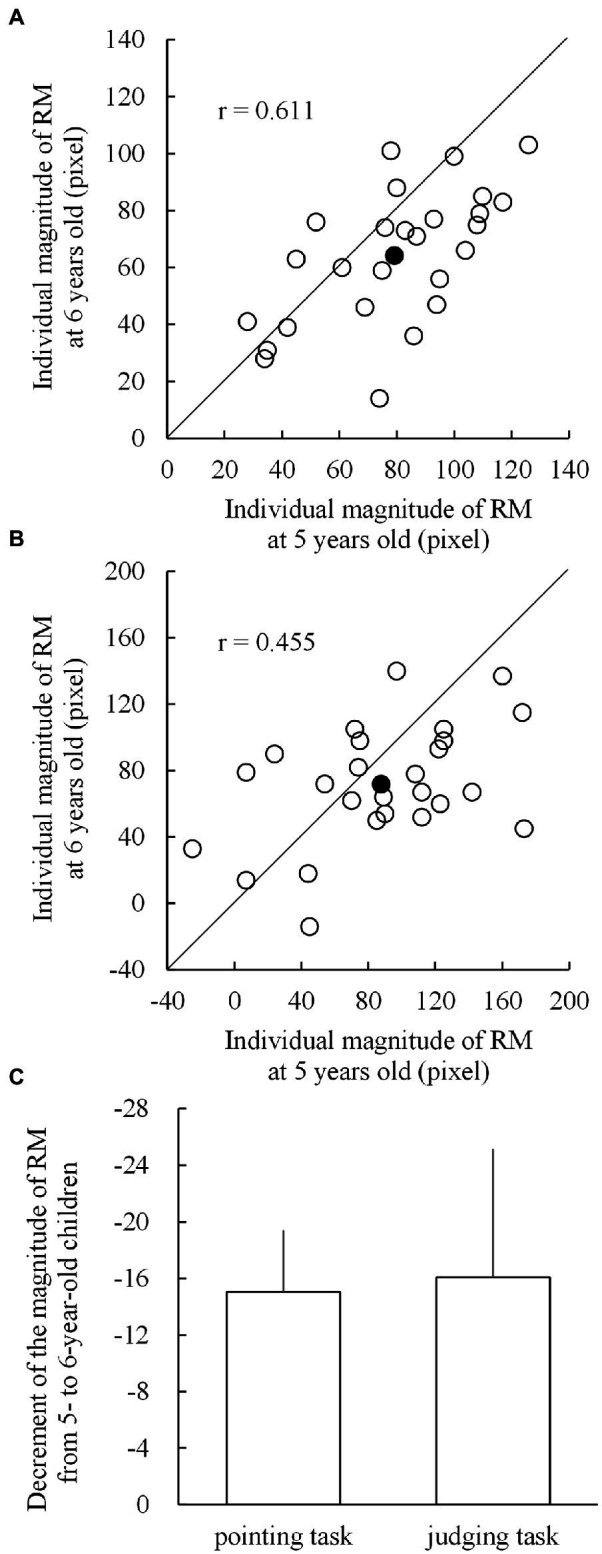
Scattergrams for the individual magnitudes of representational momentum (RM) at the age of 5 (horizontal axis) and 6 years (vertical axis) in **(A)** the pointing task and **(B)** the judging task. The blank circles below the diagonal lines indicate individuals who showed a decreased magnitude of RM from 5 to 6 years of age. The filled circles indicate the mean magnitudes of RM. **(C)** Shows the respective mean decrements from 5 to 6 years of age for the pointing and judging tasks, with the error bars indicating 1 standard error.

To visualize the individual developmental changes in the magnitude of RM from the age of 5 to 6 years, we plotted scattergrams of individual RMs at both ages for both pointing (Figure 4A) and judging tasks (Figure 4B). The correlations between the magnitudes of RM at the ages of 5 and 6 years for the pointing and judging tasks were 0.611 and 0.455 (*p* < 0.001 for both), respectively. Individual data plotted below the diagonal lines in Figures 4A,B indicate developmental (longitudinal) decrements in the magnitude of RM. In the pointing task (Figure 4A), 21 out of 26 children showed decrements and five showed increments, whereas in the judging task (Figure 4B), 17 children showed decrements and nine showed increments.

Individual developmental changes in the magnitude of RM were calculated by subtracting the individual magnitudes of RM at the age of 5 years from those at the age of 6 years. Thus, as shown in Figure 4C, the mean decrements for the pointing and judging tasks were − 15.0 (SD = 22.6) and − 16.1 (SD = 47.2) pixels, respectively. The two mean decrements did not significantly differ (*t*_25_ = −0.097; *p* = 0.924; Cohen’s *d* = −0.019) to each other, and both mean decrements were significantly lower than 0 (*t*_25_ = −3.386, *p* = 0.001, Cohen’s *d* = −0.664, for the pointing task; *t*_25_ = −1.737, *p* = 0.047, Cohen’s *d* = −0.341, for the judging task).

### *Ad hoc* Analyses Based on This and the Shirai et al. Study

**Figure 5:**
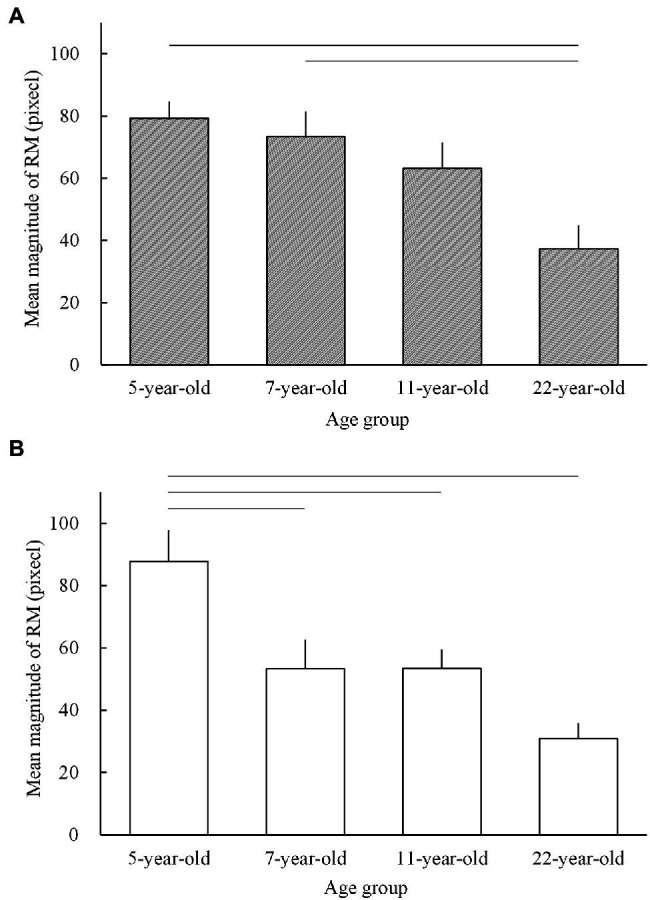
The mean magnitudes of representational momentum (RM) for 5-year-old children from the present study and 7- and 11-year-old children and 22-year-old adults from the Shirai et al. (2018) study in **(A)** the pointing task and **(B)** the judging task. The horizontal lines indicate significant differences (*p* < 0.05; see the text for the respective *p* values). The error bars present 1 standard error.

We further examined whether the magnitudes of RM obtained in this study were smaller or larger than those in older school-aged children and/or adults (Shirai et al., 2018). As mentioned in the introduction, developmental changes in the magnitudes of RM in school-aged children and adults have been examined previously (Shirai et al., 2018) using a similar method. Based on the data from this and the Shirai et al. (2018) study, the overall developmental changes in the magnitude of RM for a wide range of age groups (5.4-year-old children^4^ from this study; 7.4- and 10.8-year-old children and 22.0-year-old adults from the Shirai et al. (2018) study) are shown in Figure 5.

An *ad hoc* two-way ANOVA with a mixed-measures design (age group as a between-participant factor and task as a within-participant factor) was performed. The results showed that the main effect was significant for age group (*F*_3, 70_ = 10.486; *p* < 0.001; $\eta_{p}^{2}$ = 0.310) but not for task (*F*_1, 70_ = 2.128; *p* = 0.149; $\eta_{p}^{2}$ = 0.029), with no significant interaction between the two factors (*F*_3, 70_ = 1.839; *p* = 0.148; $\eta_{p}^{2}$ = 0.073). The simple main effect of age was significant in the pointing (*F*_3, 70_ = 6.761; *p* < 0.001; $\eta_{p}^{2}$ = 0.225; Figure 5A) and judging tasks (*F*_3, 70_ = 8.117; *p* < 0.001; $\eta_{p}^{2}$ = 0.258; Figure 5B). Subsequent multiple comparisons tests (Tukey) showed that for the pointing task, the mean magnitudes of RM were larger in 5- (mean, 79.27 pixels;^5^ SE = 5.34; *p* < 0.001) and 7- (mean = 73.31 pixels; SE = 8.02; *p* = 0.007) than in 22-year-old adults (mean, 37.19 pixels; SE = 7.70). Furthermore, the magnitude of RM in 11-year-old children (mean, 63.13 pixels; SE = 8.36) did not reach significance (*p* = 0.085) compared to that in 22-year-old adults, and no other combinations showed significance (*p* > 0.3). For the judging task, the mean magnitude of RM in 5-year-old children (mean, 87.77 pixels; SE = 10.00) was significantly larger than that in 7- (mean, 53.25 pixels; SE = 9.29; *p* = 0.028), 11- (mean, 53.38 pixels; SE = 6.08; *p* = 0.028), and 22-year-old groups (mean, 30.88 pixels; SE = 5.03; *p* < 0.001).

## Discussion

### Developmental Decrements in the Magnitude of RM From 5 Years to 6 Years of Age

Our primary concern was the developmental and longitudinal changes in the magnitude of RM among nursery school children. The main finding is shown in Figure 4, indicating that the mean developmental changes in the magnitude of RM from 5 to 6 years of age clearly showed significant decrements for the pointing and judging tasks. This suggests that the magnitudes of RM decreased with age in young children, even in 12 months, from the age of 5.4 to 6.4 years. This is consistent with the result of ANOVA on the mean difference in the magnitude of RM between 5- and 6-year-old children (Figure 3) for the pointing task but not for the judging task (*p* = 0.095). A likely reason for the insignificant age effect on the mean magnitude of RM arising in the judging task might have resulted from a relatively large variance (SD = 51.0) for 5-year-old children in the judging task compared to other SD (36.2 for 6-year-old children in the judging task, and 27.2 and 23.6 for 5- and 6-year-old children in the pointing task). In contrast, the large variance in the 5-year-old children for the judging task may not have affected the results of significant developmental decrements, shown in Figure 4C. This is because the individual decrements in the magnitude of RM were calculated per participant by subtracting the magnitude of RM in 5-year-old from that in 6-year-old children, thus resulting that the individual decrements should be free from the absolute values of the individual magnitude of RM, irrespective of whether they are large or small. In sum, the present longitudinal examination on the changes in the magnitude of RM from 5 to 6 years of age supports the developmental attenuation of the magnitude of RM, as previously reported in cross-sectional studies (Hubbard et al., 1999; Shirai et al., 2018), as a developmental feature rather than a consequence of large individual differences in RM among children.

### Overall Developmental Changes in the Magnitude of RM From Childhood to Adulthood: Comparison of This and Shirai et al. Studies

The results of the *ad hoc* ANOVA indicated that for the pointing task (Figure 5A), the magnitude of RM significantly decreased from the age of 5 and 7 years (but not in 11-year-old children) to the age of 22 years, whereas for the judging task (Figure 5B), the magnitude of RM significantly decreased from the age of 5 years to the age of 22 years. Therefore, developmental changes in the magnitude of RM are characterized by a general *developmental decrement* from early childhood to adulthood for both pointing and judging tasks. The present finding that the magnitude of RM for the pointing task was greater in early childhood than in adulthood is consistent with the findings of a previous study (Hubbard et al., 1999). However, this was inconsistent with the findings for the judging task (Futterweit and Beilin, 1994), which did not significantly differ between children and adults. The reason for the inconsistency in the judging task is unclear. Nevertheless, both the Futterweit and Beilin (1994) study and the present study showed a relatively smaller effect size of the age factor for the judging task, which may lead to weaker statistical power in examining age effects for the judging task than for the pointing task. This could thus result in equivocal statistical results, either significant or insignificant, for the judging task between the present study and the Futterweit and Beilin (1994) study. In the present study, our *ad hoc* examination showed a significant difference in the magnitudes of RM between young children and adults for the judging task (and pointing task), and our examination of the developmental decrements in the magnitude of RM from 5 to 6 years old also showed significant decrements for the judging task (and also the pointing task). In sum, the findings in the present and Shirai et al.’s studies provide evidence for the developmental “decrement” in the magnitude of RM from early childhood to adulthood for both pointing and judging tasks, with developmental changes in RM being somewhat equivocal (i.e., linear and/or nonlinear changes) among younger and older children (5–11-year-old children), which may differ between pointing and judging tasks. The reasons for the differences between the pointing and judging tasks are discussed in the latter part of the discussion section.

### Causal Factor(s) Underlying the Greater Magnitude of RM in Early Childhood and Developmental Decrements

The reason(s) for greater magnitudes of RM in early childhood than in adulthood is unclear. In this section, we discuss three speculations regarding the likely reasons for the greater magnitudes of RM in younger children than in adults. Hubbard et al. (1999) attempted to explain their results (i.e., the magnitude of RM in the pointing task is greater in children than in adults) because RM may generally result from the processes of an “analog” representation, rather than that of a “propositional” representation (Kelly and Freyd, 1987), and younger children may tend to more rely on analog than on propositional representation. Therefore, younger children may have a greater magnitude of RM than adults do. However, recent neuroscientific studies (Falkenberg et al., 2014; Joshi and Falkenberg, 2015; Gilmore et al., 2016) have shown that children in late childhood have lower sensitivity to events of smooth visual motion than adults. These findings may not be fully consistent with the explanation provided by Hubbard et al. (1999) regarding the analog versus propositional representation, and do not fully explain the reason for our findings that the magnitude of RM is greater in early childhood than in adulthood.

Second, we speculate that the greater magnitude of RM in younger children (5–7-year-olds) than in adults may be related to younger children’s difficulty performing RM tasks, such as pointing and judging. It is likely that task difficulty could largely differ between young children and adults, with younger children having more difficulties than adults. Younger children’s difficulties in performing RM tasks may result from some likely deficiencies, such as slow reaction times and lower sensitivity to motion events. Simple reaction times (reflecting neural processing times) are longer in younger children than in adults and sharply decrease (or shorten) from the age 4–10 years; thereafter, simple reaction times gradually decrease until adulthood (Dykiert et al., 2012; Maruta et al., 2017). Furthermore, younger children are likely to have a relatively lower sensitivity to motion events than adults (Falkenberg et al., 2014; Joshi and Falkenberg, 2015; Gilmore et al., 2016). A likely functional role of RM is to compensate for neural processing delays that arise in the perceptual processes (i.e., from visual input to cortical representation) of motion events (Kerzel and Gegenfurtner, 2003; Hubbard, 2005). To compensate for the neural processing delays arising under motion events, younger children’s slower reaction times and lower sensitivity to motion events might be a great disadvantage in their ability to adequately perform RM tasks. In alleviating such a younger children’s disadvantage in compensating for the neural processing delays for motion events, a greater magnitude of RM might be a great benefit (thus needed) in younger children than in adults. This hypothesis should be examined in future studies.

We also speculate about the innate nature of RM. The present study showed that the magnitude of RM in younger children was greater than that in adults and decreased with age, indicating developmental decrements. This finding is inconsistent with those of studies that reported a large magnitude of RM in sports experts versus novices (Blättler et al., 2010, 2011, 2012; Gorman et al., 2011, 2012; Nakamoto et al., 2015; Jin et al., 2017; Anderson et al., 2019; Chen et al., 2021). The large magnitude of RM in sports experts is well explained by their long-term training and experience in seeing relatively fast-moving events in sports (Gorman et al., 2011, 2012; Jin et al., 2017; Chen et al., 2021). Considering the findings of studies on the RM of sports experts and those of the present study, the basic nature of RM may be twofold: the acquired nature from long-term learning or experience of motion and/or fast-moving events and the innate nature based on ontogenetic processes. The innate nature of RM is speculated to develop in the early stage of growth or during the ontogenesis process as a fundamental visual function to compensate for a likely neural delay arising in neural processes (e.g., see Kerzel and Gegenfurtner, 2003; Hubbard, 2005). Furthermore, it may be gradually suppressed or degraded through growth by some developed higher or cognitive functions, thus overcoming or suppressing the basic innate nature of RM. This view is speculative and does not fully explain the reason(s) for the greater magnitudes of RM in early childhood than in adulthood, as well as its developmental decrement from early childhood to adulthood. This should be examined further in future studies.

### Difference in the Developmental Changes of the Magnitude of RM for the Pointing and Judging Tasks

The present and Shirai et al. (2018) studies indicated that the developmental changes in the magnitude of RM during early and/or late childhood differed for pointing and judging tasks (Figures 5A,B). For the pointing task, no significant difference in the magnitude of RM was observed for early and late childhood, whereas for the judging task, 5-year-old children showed a greater magnitude of RM than the 7- and 11-year-old children. These different features of developmental changes in the magnitude of RM for pointing and judging tasks can be speculated to result from the different nature of underlying processes, such as perception, memory, and motor aspects in pointing and judging tasks (Kerzel et al., 2001; Kerzel and Gegenfurtner, 2003; Müsseler et al., 2008).

For the judging task used in the present study, participants should compare the short-term memory representation of the vanishing point of a “moving” stimulus with the perceived position of a “probe” stimulus presented 400 ms after the moving stimulus had vanished (the probe stimulus remained stationary until the participant made a verbal response). Such a comparison process involving the short-term memory and perception is generally thought of as a part of working memory system (Buss et al., 2018), which develops in early childhood, such as 3- to 6-year-old ages. It is therefore likely that the comparison of the short-term memory representation of the vanishing point of a moving stimulus with the perceived location of the probe stimulus may have been somehow difficult for young children compared to adults. In contrast, for the pointing task, no probe stimulus was presented after the moving stimulus had vanished. Thus, the participants simply pointed at the vanishing point with their finger according to the perception, or short-term memory, of the vanishing point of a moving stimulus, with no comparison between the memory representation of a moving stimulus and perception of a probe stimulus. Such different underlying processes for the pointing and judging tasks may have caused the different features of the developmental changes in RM for the pointing and judging tasks, although it is far from clear how the different nature of the relevant processes of perception and memory would cause different developmental features for the pointing and judging tasks. This should be further examined in future studies.

For another view point on the neural processes underlying RM, the judging task is a type of “seeing” task that involves both visual perception and mental judgment aspects. In contrast, the pointing task is a “seeing” plus “motor action” task, which involves the processing of both visual perception (plus judgment) and motor action and is thought to be primarily underpinned by the dorsal stream of the visual systems (Goodale and Milner, 1992). The likely different processing natures involved in the two tasks may have caused the resultant difference in features of the developmental changes in the magnitude of RM. Furthermore, the concept of “analog” and “propositional” representation, which Hubbard et al. (1999) proposed in their explanation of the greater RM magnitude in childhood than in adulthood (as shown at the previous part of the “Discussion”), could be a causal factor for the different features for the pointing and judging tasks regarding developmental changes in the magnitude of RM. Nevertheless, it is unclear how the different processing nature of the pointing and judging tasks, such as “seeing” versus “acting,” “analog” versus “propositional” representation, and/or “perception” versus “memory,” contribute to the different developmental changes in the magnitude of RM for the two tasks and warrants further investigation in future studies.

### A Methodological Issue: Effectiveness of Subtraction Method for Estimating True RM

Our results for the individual displacement data (Figures 1, 2) clearly show that the mean displacements under the immediate-vanishing condition were much larger than those under the delayed-vanishing condition for the pointing (Figure 1C) and judging (Figure 2C) tasks. Regarding individual displacements (Figures 1A,B for the pointing task; Figures 2A,B for the judging task), large variations among participants were observed for the immediate- and delayed-vanishing conditions. Therefore, the subtraction method was used to calculate the true individual magnitudes of RM without individual biases. If raw displacements (i.e., under the immediate-vanishing condition) were used as the magnitude of RM rather than those calculated using the subtraction method, some possible individual biases could have affected the resultant magnitudes. Regarding possible individual biases, for example, Pellizzer and Hauert (1996) showed that young children (6–8 years old) tended to overshoot a target in a pointing task in the right visual field; thus, some individual biases could often occur with respect to either visual perception, judgment, or motor action. Therefore, it is necessary to eliminate or minimize individual biases when estimating the true magnitudes of RM. Shirai et al. (2018) presented the effectiveness of the subtraction method for calculating the true RM; moreover, this is clearly replicated in the present study. Therefore, the subtraction method should be used when calculating the magnitude of RM to eliminate or minimize any individual biases with respect to perceptual, judging, and/or motor processes.

## Conclusion

The main finding of the present study is the significant decrease in the mean magnitude of RM from 5–6-year-old children for the pointing and judging tasks, although the mean magnitude of RM *per se* was significantly greater in 5-year-old versus 6-year-old children in only the pointing task. Our *ad hoc* analyses using the results of this and the Shirai et al. (2018) studies showed that the magnitude of RM was significantly greater in early childhood than in adulthood for the pointing and judging tasks. Therefore, the present findings suggest that the magnitude of RM significantly decreases from early childhood to adulthood, with significant developmental decrements occurring for both tasks. As a methodological issue, the subtraction method for calculating the magnitudes of RM would be useful for eliminating or minimizing any individual biases possibly involved in the pointing and judging tasks.

## Data Availability Statement

The original contributions presented in the study are included in the article, further inquiries can be directed to the corresponding author.

## Ethics Statement

The studies involving human participants were reviewed and approved by The Ethics Committee of the National Institute of Fitness and Sports in Kanoya, Japan. Written informed consent to participate in this study was provided by the participants’ legal guardian/next of kin.

## Author Contributions

KI developed the study conception, experimental design, and software programs used for the experiments. SM contributed to conducting experiments, data collection, statistical analyses, and wrote the first draft of the manuscript. HN and NS contributed to revision of statistical analyses and *ad hoc* analyses. All authors contributed to organizing the discussion section, manuscript revision, and the submitted version of the manuscript.

## Funding

This research was supported by Grant-in-Aid for Scientific Research from the JSPS (Grant nos. 17H00875 and 21K18562) to KI.

## Conflict of Interest

The authors declare that the research was conducted in the absence of any commercial or financial relationships that could be construed as a potential conflict of interest.

## Publisher’s Note

All claims expressed in this article are solely those of the authors and do not necessarily represent those of their affiliated organizations, or those of the publisher, the editors and the reviewers. Any product that may be evaluated in this article, or claim that may be made by its manufacturer, is not guaranteed or endorsed by the publisher.

## Footnotes

^1^The sample size was calculated using GPower version 3.1.9.2 with predetermined input parameters: effect size $\eta_{p}^{2}$= 0.131 (*f* = 0.387), from the effect size for the age effect in the judging task reported by Shirai et al. (2018); *α* = 0.05; power (1 – *β*) = 0.95; number of groups = 1; number of repetitions = 2; and correlation between repetitions = 0 (it was unknown, although this could presumably be larger than zero). This resulted in a sample size of 46. Thus, at least 23 participants (46/2) were required.

^2^The size of the visual angle was 1.68 times larger in the present study than in that by Shirai et al. (2018) because of different viewing distances for the two studies. In the present study, the viewing distance was approximately 30–42 cm (mean, 36 cm) at the center and edge of the 27-inch touch-sensitive LCD screen versus 57–64 cm (mean 60.5 cm) in the Shirai et al. (2018) study. For convenience, we calculated each visual angle simply based on the estimated viewing distance of 36 cm, although viewing distances varied according to participant body size or arm length.

^3^Based on the data of Shirai et al. (2018), the effects of task order (pointing or judging task first performed) and vanishing condition order (immediate- or delayed-vanishing condition performed first for pointing and judging tasks, which resulted in four vanishing condition orders) on the magnitudes of RM were calculated. Our results showed no significant order effects for either the task order or vanishing condition order in younger (7-year-old) and older (11-year-old) children. Therefore, in the present study, we fixed the order of tasks and vanishing conditions to reduce the likelihood of difficulty performing pointing and judging tasks. To determine the effect of task order on the pointing and judging tasks, we performed ANOVA of task order and age group. Our results showed no significant main effect of task order (*F*_1,28_ = 1.713; *p* = 0.201) or age (*F*_1,28_ = 0.771; *p* = 0.387), with no significant interaction between task order and age (*F*_1,28_ = 0.200; *p* = 0.658), in the pointing task. Similarly, no significant main effect of task order (*F*_1,28_ = 0.030; *p* = 0.863) or age (*F*_1,28_ = 0.00001; *p* = 0.991), with no significant interaction between task order and age (*F*_1,28_ = 0.057; *p* = 0.812), was found in the judging task. Similar to the task order, the vanishing condition order had no significant effect on the magnitude of RM. Specifically, our results showed no significant main effect of vanishing condition order (*F*_3,24_ = 2.386; *p* = 0.094) or age (*F*_1,24_ = 1.206; *p* = 0.283), with no significant interaction between vanishing condition order and age (*F*_3,24_ = 0.649, *p* = 0.591), in the pointing task. Similarly, no significant main effect of vanishing condition order (*F*_3,24_ = 0.294; *p* = 0.829) or age (*F*_1,24_ = 0.025; *p* = 0.876) or no significant interaction between vanishing condition order and age (*F*_3,24_ = 0.888; *p* = 0.462) was found in the judging task.

^4^For the *ad hoc* ANOVA, age group was managed as a between-participant factor based on four groups, namely, 5-, 7-, and 11-year-old children and 22-year-old adults. The 6-year-old data obtained in the present study were excluded from the *ad hoc* analyses because of the nature of repeated measures data (the 6-year-old children had already performed the experiment at 5 years of age).

^5^We generally reported SD rather than SE elsewhere in the "Results" section, whereas Shirai et al. (2018) had reported SE instead of SD. Therefore, we used SE when comparing data between this and the Shirai et al. (2018) study.
